# How infant‐directed actions enhance infants’ attention, learning, and exploration: Evidence from EEG and computational modeling

**DOI:** 10.1111/desc.13259

**Published:** 2022-04-07

**Authors:** Marlene Meyer, Johanna E. van Schaik, Francesco Poli, Sabine Hunnius

**Affiliations:** ^1^ Donders Institute for Brain, Cognition and Behavior Radboud University Nijmegen Nijmegen The Netherlands; ^2^ Department of Psychology University of Chicago Chicago USA; ^3^ Behavioural Science Institute Radboud University Nijmegen Nijmegen The Netherlands

**Keywords:** attention, computational modeling, EEG, infants, infant‐directed actions, surprise, theta oscillations, variability

## Abstract

When teaching infants new actions, parents tend to modify their movements. Infants prefer these infant‐directed actions (IDAs) over adult‐directed actions and learn well from them. Yet, it remains unclear *how* parents’ action modulations capture infants’ attention. Typically, making movements larger than usual is thought to draw attention. Recent findings, however, suggest that parents might exploit movement variability to highlight actions. We hypothesized that variability in movement amplitude rather than higher amplitude is capturing infants’ attention during IDAs. Using EEG, we measured 15‐month‐olds’ brain activity while they were observing action demonstrations with normal, high, or variable amplitude movements. Infants’ theta power (4–5 Hz) in fronto‐central channels was compared between conditions. Frontal theta was significantly higher, indicating stronger attentional engagement, in the variable compared to the other conditions. Computational modelling showed that infants’ frontal theta power was predicted best by how surprising each movement was. Thus, surprise induced by variability in movements rather than large movements alone engages infants’ attention during IDAs. Infants with higher theta power for variable movements were more likely to perform actions successfully and to explore objects novel in the context of the given goal. This highlights the brain mechanisms by which IDAs enhance infants’ attention, learning, and exploration.

## Graphical abstract

We investigated whether variability in movement amplitude rather than higher amplitude is capturing infants’ attention during infant‐directed actions (IDAs). Using EEG, we measured 15‐month‐olds’ brain activity while they were observing action demonstrations with normal, high, or variable amplitude movements. Infants’ theta power (4–5 Hz) in fronto‐central channels was significantly higher, indicating stronger attentional engagement, in the variable compared to the other conditions. Computational modelling showed that infants’ frontal theta power was predicted best by how surprising each movement was. This highlights the brain mechanisms by which IDAs enhance infants’ attention, learning, and exploration.

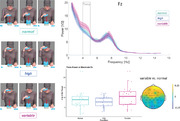

## 1 INTRODUCTION

When showing novel actions to their infants, such as how to use a new rattle or how to stack blocks, parents tend to modify their movements. Infant‐directed actions (IDAs) differ from adult‐directed actions (ADAs) in movement amplitude and speed, in the number of repetitions (Brand et al., 2002; van Schaik et al., 2020), in the number of toy exchanges, and in the duration of action effect demonstrations (Brand et al., 2002; Brand et al., 2007; van Schaik et al., 2020). What is the function of this phenomenon that seems counterintuitive at first, as parents are demonstrating a version of the action not identical to the one to be learned by the infant? The key to understanding this phenomenon likely is the functional role IDAs have for infants’ attention and learning. Like infant‐directed speech (ManyBabies Consortium, 2020), IDAs enhance infants’ attention to the demonstrated action (Brand & Shallcross, 2008; Koterba & Iverson, 2009) and facilitate action learning (Schreiner et al., 2020; Williamson & Brand, 2014). Evidence for such attentional enhancement comes from looking time studies with infants and young children. Brand and Shallcross (2008) tested 6‐ to 8‐month‐olds and 11‐ to 13‐month‐olds in a preferential looking paradigm, presenting them with IDAs and ADAs. Their results show that infants of both age groups preferred looking at IDAs compared to ADAs. Similarly, Koterba and Iverson (2009) investigated the looking behavior of 8‐ to 10‐month‐old infants when observing their caregiver demonstrate actions on novel toys. In this study, parents were instructed to demonstrate an action with high or low amplitude movements and repeat it or not. Infants looked longer at IDAs if they were performed with high amplitude movements, were repeated or had high amplitude movements which were repeated, compared to non‐repeated low amplitude movements.

In addition to enhanced attention during IDAs, several studies have demonstrated that IDAs affect infants’ action exploration and learning (Koterba & Iverson, 2009; van Schaik et al., 2020; Williamson & Brand, 2014). For instance, Williamson and Brand (2014) assessed the imitation performance of 2‐year‐old children; a baseline group who did not see any action demonstrations, an ADA group and an IDA group. Children who had IDA demonstrations were more likely to perform the demonstrated action compared to the ADA and baseline groups. While this body of research suggests that IDAs play an influential role in infants’ attention to and learning of actions, it is unknown *how* IDAs capture infants’ attention and guide their learning.

### 1.1 How do IDAs draw infants’ attention?

One prototypical feature associated with IDAs is the use of larger movements than usual. One possibility is, therefore, that IDAs draw infants’ attention because they are larger than normal actions. Studies in which adults were instructed to make large movements when demonstrating an action found attentional and learning effects in infants (Koterba & Iverson, 2009; Williamson & Brand, 2014). However, movement amplitude was manipulated together with other features, like repetition, emotional engagement, and proximity to the infant (Koterba & Iverson, 2009; Williamson & Brand, 2014). Two other findings render it less likely that large movements are the driving force of infants’ attention during IDAs. That is, Koterba and Iverson (2009) saw increased looking time to IDAs performed repeatedly with small amplitude compared to a control condition and large movements were found to be dependent on the action at hand rather than universal across actions (van Schaik et al., 2020). While making large movements remains a possible factor underlying infants’ attention to IDAs, recent findings suggest another potential driving factor: variability in movement.

**RESEARCH HIGHLIGHTS**

- 15‐month‐olds watched actions with normal, high, and variable movement amplitude
- Infants show increased frontal theta power when actions contain variable movements
- Frontal theta during variable movements relates to infants’ subsequent learning and exploration
- Computational modelling suggests that surprise, induced by variability, drives theta

In a study by Fukuyama et al. (2015) parents were found to dynamically modulate the variability in their IDAs dependent on their children's behavior. More specifically, the authors measured mothers’ movement kinematics and their 11‐ to 13‐month‐olds’ behavioral performance during a dyadic interaction in which mothers demonstrated a cup‐nesting action. Mothers increased the variance in their movements after their infant had performed irrelevant actions with the cups in between demonstrations and decreased their movement variance after their infant performed the target actions. This suggests that movement variability might be exploited by parents to direct their infants’ attention to the demonstrated action when needed. Performing movements that deviate from the usually small and efficiently performed movements parents make might represent such a natural variation as well. Thus, movement variability and amplitude remain confounded in everyday life. Furthermore, this role of variability is supported by findings from other lines of research, such as statistical learning work which suggests that infants pay more attention to variable, less predictable input (Johnson & Munakata, 2005; Tummeltshammer & Kirkham, 2013). In sum, instead of solely large movement amplitudes, variability in movements could be responsible for capturing infants‘ attention, thereby influencing learning and exploration in IDAs.

### 1.2 The current study

In this EEG study, we investigated the roles of variability and movement amplitude in enhancing infants’ attention to IDAs. We hypothesized that variability in movement attracts infants’ attention more than large movements per se. We also investigated the relation between infants’ attention to variable movements and their learning.

So far, studies on IDAs have relied on looking time measures as proxy for infants’ attention. In the current study, we made use of a neural indicator of infants’ attention which allows for a more sensitive measure which does not depend on behavioral changes. More specifically, we examined modulations in frontal theta band oscillations. Theta band activity in frontal brain regions has previously been linked to top‐down and bottom‐up attention in infants (Begus et al., 2015; Orekhova et al., 2006), young children (Meyer et al., 2019), and adults (Clayton et al., 2015). Frontal theta power has been proposed to signal the need for cognitive control (Cavanagh & Frank, 2014), often elicited by attention capturing events. Frontal theta band modulations are also associated with memory processes (Begus et al., 2015; Jensen & Tesche, 2002) and are thought to reflect infants’ learning of new information (Begus & Bonawitz, 2020). For instance, findings on neural processing of infant‐directed speech provide evidence for an increase in infants’ frontal theta power when listening to infant‐directed speech compared to control conditions (Orekhova et al., 2006; Zhang et al., 2011). Thus, frontal theta band power is a promising neural measure to assess infants attentional processing during IDAs.

Our experimental set‐up consisted of a demonstration and an exploration phase. We measured 15‐month‐old infants’ brain activity while they observed action demonstrations executed with normal, high, and variable movement amplitudes. After the demonstration phase, infants had the opportunity to perform the actions themselves. To investigate how the different conditions affected infants’ attentional processing, we compared infants’ theta power (4–5 Hz) in fronto‐central midline channels (Fz, FCz, Cz) between conditions, controlling for multiple comparisons. In addition, we computed a linear regression model to examine the link between infants’ attention as reflected by their frontal theta power to variable amplitude movements and their exploration and learning behavior. As an exploratory analysis, we further investigated which aspect of the movement variability might be driving any potential effects. Variable movements are both more surprising and more complex. To disentangle whether surprise or complexity better described infants’ frontal theta power, we additionally used a computational modelling approach.

## 2 MATERIAL AND METHODS

### 2.1 Participants

In the final sample 23 infants (10 girls) are included. Infants’ mean age was 15.9 months (range = 15.5–16.5 months). We chose this age on the basis of previous research on IDAs showing that caregivers modulate their movements towards infants of this age and showing that around this age infants are capable of imitating basic actions, an important aspect for the learning part of this study (van Schaik et al., 2020). Another 24 infants were tested of which 12 had to be excluded due to an error in the automatized randomization, five were excluded because of insufficient EEG recording quality and another seven participants did not contribute sufficient artifact‐free trials to the analysis (see *EEG analysis* for details). A sample size of 20 participants was estimated for a power of 0.85 for the main analysis, a repeated measures ANOVA with three within‐subjects conditions (assuming a medium effect size f of 0.25, an alpha level of 0.05 and a correlation among repeated measures of 0.7). Anticipating an approximate 40% drop out rate, common in infant EEG studies, we tested 35 participants. Note that the additional 12 participants who needed to be excluded due to a randomization error were replaced. Families who participated in the study came from a middle‐sized Dutch city and were recruited from a database of families volunteering in developmental research. The current study adheres to the Declaration of Helsinki and was approved by the local ethics board. Parents gave written consent to their infant's participation in the study. As compensation for their travels and time, families could choose to receive either two children's books, a children's book and 10 euros, or 20 euros.

### 2.2 Stimuli

**FIGURE 1:**
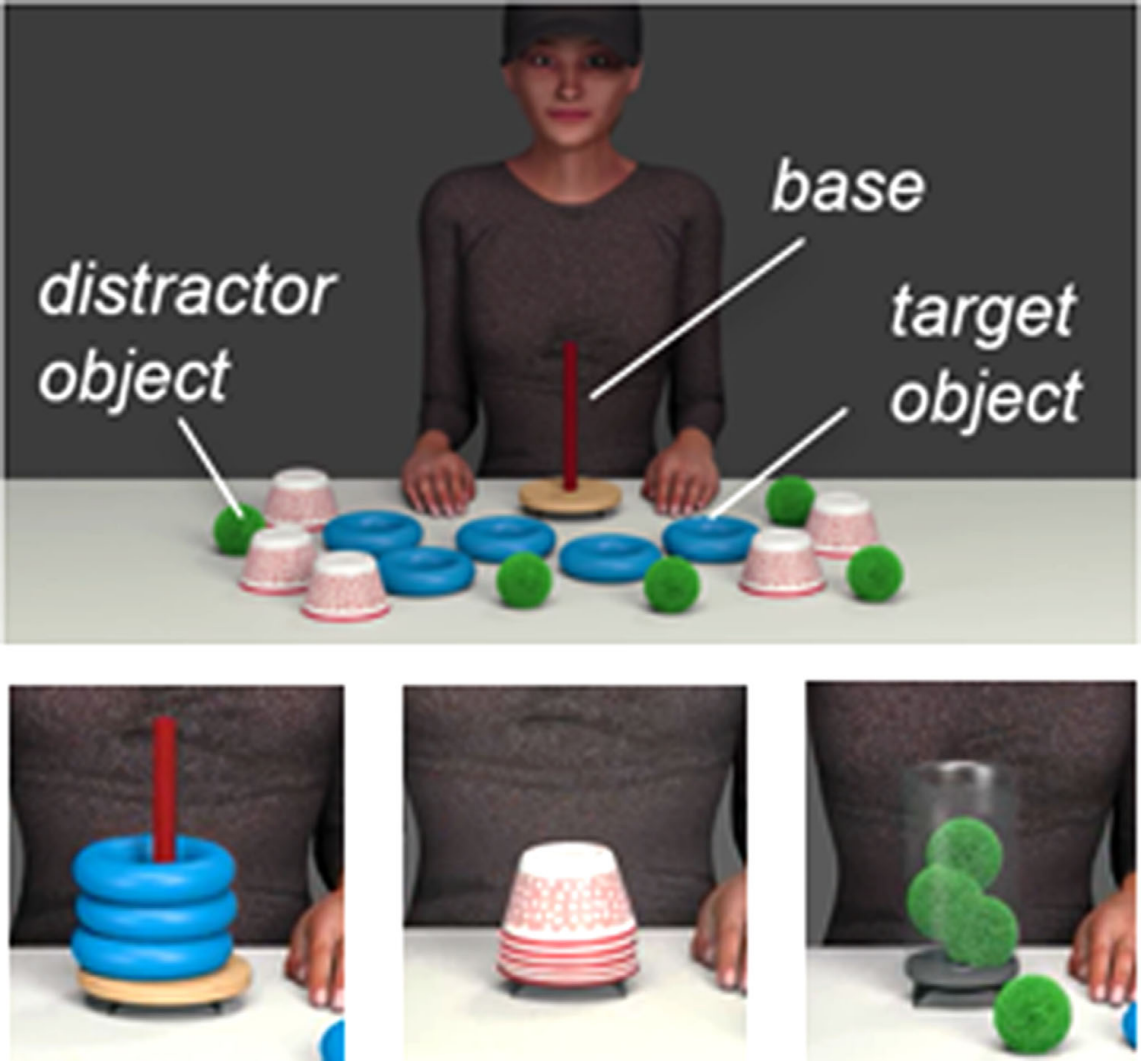
Top: Example of the video stimuli showing an avatar with a goal base (here: peg) surrounded by corresponding target objects (here: rings) and distractor objects (here: balls and cups). The avatar acted on the goal base using the target objects. Bottom: Three different goal states representing the actions, that is, stacking rings on a peg, building a tower with cups and putting balls in a container, were each shown in one condition (normal, high, variable), counterbalanced across participants

For this study, we created avatar stimuli based on motion‐tracking recordings of an adult model (see Figure 1). The advantage of using avatars and a virtual environment instead of video recordings was the precise manipulation of movement amplitude in the stimuli. That is, it allowed us to create instances of low and high amplitude movements with human kinematic features that could be combined into different stimulus movies. Using a Qualisys motion‐tracking system (a Qualisys Oqus 5 + system with seven infrared cameras for motion‐tracking and one video camera) we recorded the movements of an adult model performing three different actions; stacking rings on a peg, building a tower with cups and putting balls in a bucket. For each of these actions, the model reached out to, picked up, and moved the target object (e.g., ring) to the corresponding goal base (e.g., peg). The model performed the same action five times, for instance putting five rings on the peg. Crucially, once converted to avatar stimuli, we manipulated the amplitude of the goal‐directed movements which preceded the final step of each action (e.g., the amplitude of lifting the ring to put it on the peg), resulting in two versions of each action, one with a normal amplitude and one with a high amplitude.

**FIGURE 2:**
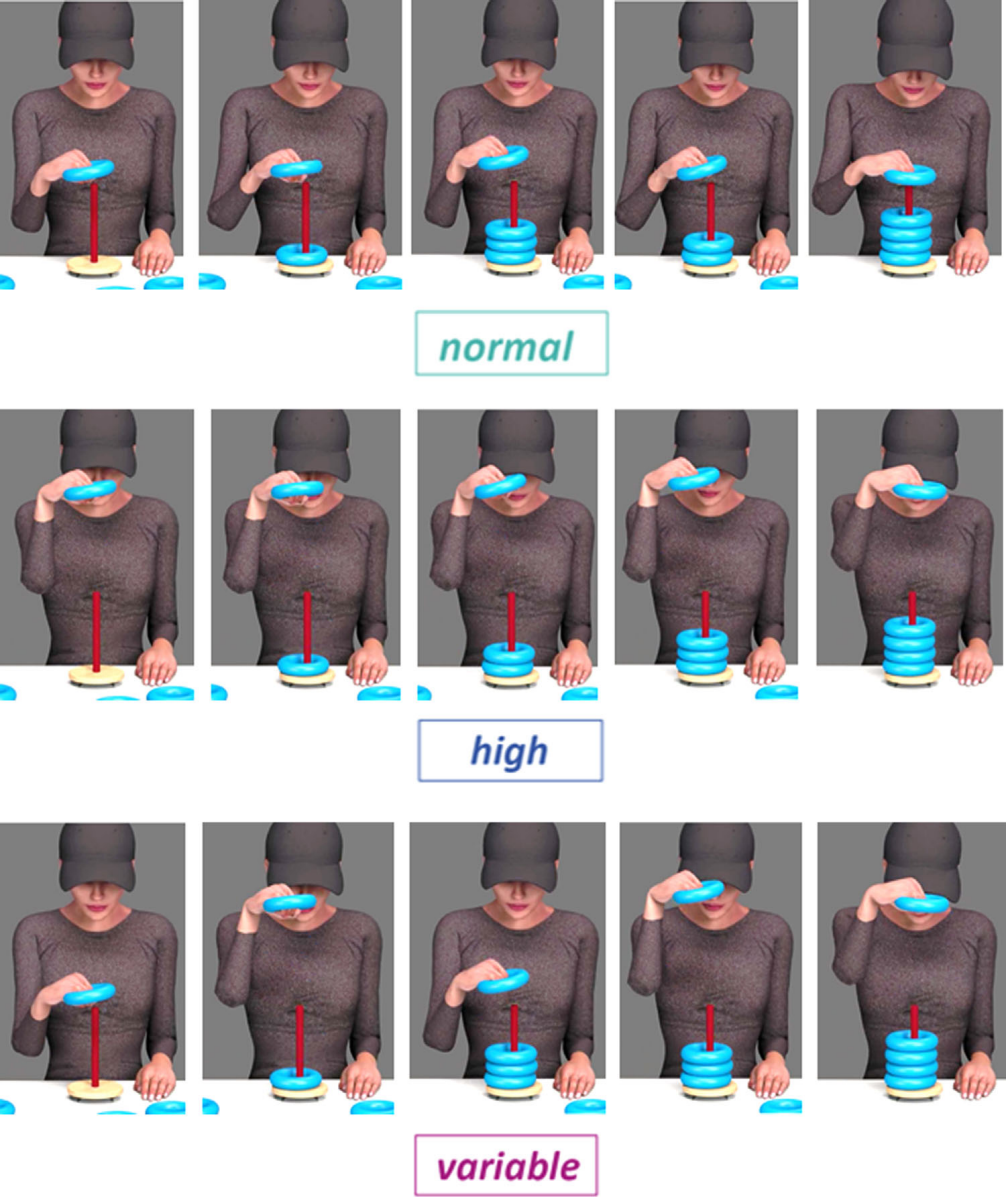
Illustration of the three conditions of interest. In the normal amplitude condition (top) the action was demonstrated with normal amplitude movements for five subsequent times. In the high amplitude condition (middle) the action was demonstrated with high amplitude movements for five subsequent times. In the variable amplitude condition (bottom) the order of high and normal amplitude movements was varied during action demonstration. It was counterbalanced between participants which action (acting on rings, balls, pegs) was associated with which condition (normal, high, variable)

From this manipulation, we made stimulus videos in three different conditions (see Figure 2) with five normal amplitude movements, five high amplitude movements, and five variable amplitude movements for each action type (using balls, rings, cups). We created the variable condition by combining the high and normal amplitude movements into different orders (e.g., a sequence of normal, high, normal, high, high; four pseudo‐randomized sequences, counterbalanced across participants).

Each stimulus video with five movements lasted for about 32 s including an initial 2 s still frame phase followed by the five goal‐directed movements of one action type. The avatar was shown with a cap that covered the upper half of the face to ensure infants would pay attention to the action rather than the actor's face and gaze. The initial visual scene of each experimental video included the model making eye‐contact and sitting at a table with all five target and ten distractor objects as well as the specific goal base in front of her (see Figure 1). The experimental videos were silent. The objects and bases used in the stimuli were also available for infants to explore in real life at the test session (see *Procedure* for details).

Additionally, we created two other types of video stimuli, intro videos and peekaboo videos. A short intro videos were made in which the infant was greeted by the actor saying "Hey, baby". The intro video lasted for about 2 s and preceded each of the experimental videos. Besides this, peekaboo videos were recorded with the actor playing the classic peekaboo game by hiding her face behind her hands for 1–4 s. The peekaboo videos served as attention getters between trials. Together the intro, the experimental and the peekaboo videos made up blocks of trials as described in more detail in the *Procedure* section.

### 2.3 Procedure

Parent and infant were invited to the lab for a combined EEG and behavioral testing session. At the beginning of the session, parents were informed about the procedure by one experimenter (E1) while another experimenter (E2) played with the infant. This introduction also allowed the infant to become familiarized with the new environment. After parents gave written consent for their infant's participation in this study, one of the experimenters fitted the infant with a 32 active electrode EEG cap (actiCap, Brain Products GmbH, Germany) while the other experimenter distracted the child with bubbles. The EEG cap was arranged in the standard 10‐20 system with the online reference electrode at the left mastoid. We strived to get impedances of all electrodes below 60 kΩ. To ensure that all electrodes touched the infant's head and to prevent infants from reaching and pulling the electrode cables, the experimenter additionally placed a tubular elastic bandage (Surgilast) over the EEG cap and electrodes.

**FIGURE 3:**
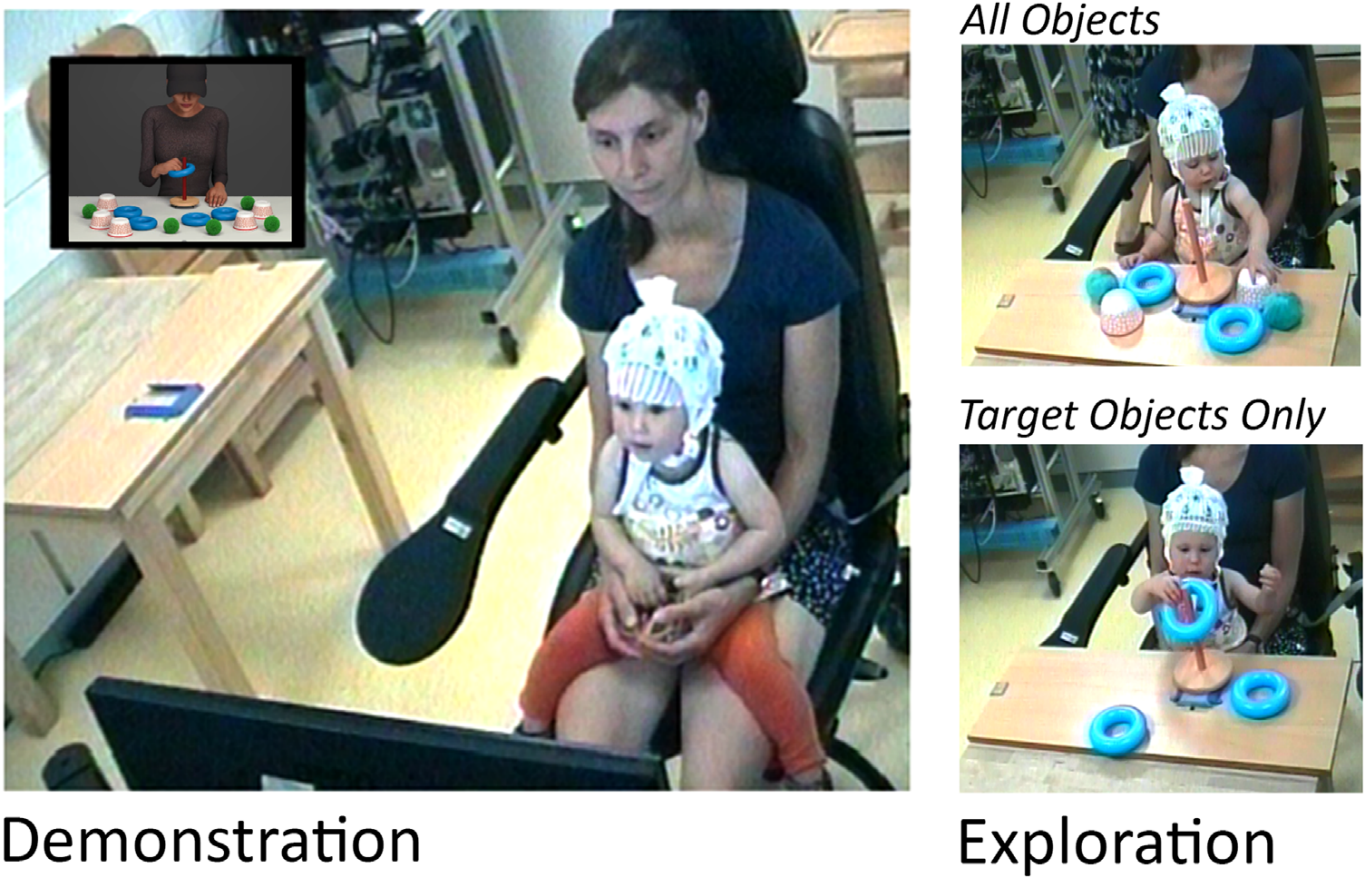
Illustration of the experimental set‐up. Left: Parent and infant participant during the demonstration phase in which novel actions are displayed using videos (see computer screen inlay in the top left corner). Infants’ neural activity was measured during this phase using a 32 active channel EEG system. Right: Parent and infant participant during the exploration phase for which infants’ behavior was video‐coded offline. Top right: During the exploration phase, first each goal base (here: peg) was presented with all objects (balls, rings, cups). Bottom right: After the availability of all objects, each goal base was presented with only the corresponding target objects (here: rings)

Once the EEG cap was prepared the experimenters accompanied parent and infant into an electronically shielded EEG testing booth. Parents were then seated in front of a screen (about 60cm distance) with their infant on their lap (see Figure 3). When parent and infant were seated comfortably, the experimenters left the room and the experimental testing session started. Infants’ EEG was amplified using a BrainAmp DC EEG amplifier, digitized at 500 Hz and recorded with a band‐pass filter of 0.1 to 200 Hz using BrainVision Recorder software (Brain Products GmbH, Gilching, Germany). Additionally, the entire testing session was video recorded for later offline coding of the infant's behavior. The experiment consisted of two testing phases: the demonstration phase and the exploration phase as illustrated in Figure 3.

During the demonstration phase, infants were presented with three actions, each shown in one of the three conditions (normal, high, and variable). Across subjects we counterbalanced which action type (acting on balls, cups, rings) was demonstrated in which condition. Each experimental video (including five goal‐directed movements) was presented in four blocks making up a total of 20 goal‐directed movements per condition. Each experimental video was preceded by a 1‐s fixation cross (white cross on a grey screen) and the intro video to orient the infant to the screen. Each block contained all three experimental videos in pseudo‐randomized order (i.e., one normal, one high, and one variable), followed by a peekaboo video. After the last block, all four peekaboo videos were played (with 1–4 s of hiding the actor's face) before commencing the exploration phase. All stimuli were presented using Presentation Software (Neurobehavioral Systems, Albany, CA) and the timing of the stimuli was automatically time‐locked with the EEG signal.

After the demonstration phase, the EEG recording was stopped, and both experimenters entered the test booth again to present infants with the objects previously shown. In this exploration phase, infants had the opportunity to act on the objects themselves. The exploration phase had two parts, the All Objects part and the Target Objects Only part. First, each of the three goal bases was presented with two target and four distractor objects (All Objects part). For instance, the peg (goal base) was presented with two rings (target objects), two balls and two cups (distractor objects) reachable for the child (see Figure 3, top right). After all three goal bases were presented in the All Objects part (order counterbalanced), each of the three goal bases was again presented one‐by‐one (order counterbalanced), but this time only with the three corresponding target objects (i.e., Target Objects Only part; see Figure 3 bottom right).

To start each exploration trial (i.e., for both All Object and Target Objects Only parts), E1 first mounted the target base in the middle of the table in front of the child. Then E1 and E2 simultaneously placed all respective objects to the left and right of the base, such that the infant had access to all objects at the same time. Each exploration trial ended after 1 min. In total, six exploration trials were conducted, three in the All Objects and three in the Target Objects Only part for which infants had 1 min each to explore. After the testing session ended, the experimenters took the EEG cap off the infant and parents were informed about the purpose of the study.

### 2.4 EEG data analysis

EEG data analysis was conducted using MATLAB (Mathworks, Inc.) and the open‐source toolbox FieldTrip (Oostenveld et al., 2011). The EEG data were time‐locked to the moment the goal of the action was reached and segmented into trials of 1 s prior to and 500 ms following this time. The timing of the action goal was defined as follows in the stimuli: the first frame in which the ball hit the bottom of the bucket or the other balls, the cup was released and touched the base or the other cups, and the ring was released and touched the base or the other rings. This 1500 ms time window was chosen to include the movement following the peak amplitude, leading up to and finishing off the action goal without overlapping with the previous or next actions. As such the window allowed us to investigate how (changes in) movement amplitude might affect neural processing of the goal‐directed action. This time window also captures six cycles of the lowest frequency of interest in our analysis (i.e., 4 Hz), allowing for robust estimates of power in this frequency. For data padding, the data were initially epoched with an additional 500 ms before and after the time window of interest.

During pre‐processing, the data were first band‐pass filtered between 1 and 30 Hz and the mean signal was subtracted from each time point to account for potential differences in offset. In the variable condition, the first trial did not offer any information about the variability in movement amplitude, and it was thus excluded. Based on video coding of infants’ gaze behavior, trials during which infants did not look at the screen were also discarded from further analysis. Then, four rounds of artifact rejection were conducted (blind to condition) and subsequently any missing channels were interpolated. There were four rounds of artifact rejection. First, epochs with large EEG artifacts were removed based on visual rejection. Second, independent‐component analysis was applied to remove components containing eye‐movement artifacts. Then, two rounds of visual artifact rejection were performed to reject any remaining trials or channels with EEG artifacts. Visual artifact rejection was performed by the first author who has extended experience in infant EEG data processing. Seven participants did not provide at least eight trials per condition and were excluded from the analyses. In the final dataset of 23 participants, an average of 15.4 trials (range: 11–20) in the normal amplitude condition, 15.6 trials (range: 11–20) in the high amplitude condition and 13.5 trials (range: 9–16) in the variable amplitude condition remained in the analysis.

Cleaned EEG data were re‐referenced to the average mastoids and linear drift was removed from the data. A fast Fourier transform was computed to estimate power for each condition between 3 and 30 Hz (for detailed settings see shared EEG data analysis scripts under *Data Availability*). Power in the theta frequency band (4–5 Hz) was then extracted. This frequency range is consistent with a band around the peak frequency (4.4 Hz) observed in previous research on infant‐directed speech (Orekhova et al., 2006) with infants of similar age. For comparison of theta power across conditions we ran repeated‐measures ANOVAs with the factor Condition (Normal, High, Variable) for a priori defined electrodes Fz, FCz and Cz, applying Bonferroni correction for conducting three tests. The EEG data processing scripts are available on GitHub (https://github.com/marlenemeyer/EEG_pipeline_variability_in_infant_directed_actions).

### 2.5 Behavioral data analysis

The exploration phase of the experiment was video coded offline using ELAN (ELAN, 2018). In the All Objects part, we coded whether the infant first touched a distractor or a target object. The number of times the target action was successfully performed was tallied and we scored whether or not infants had performed the target action successfully at least once. For the Target Objects Only part, we also coded whether the infant performed the target action at least once, and if so, how often it was performed successfully. An overview of the behavioral performance is given in Table 1. For more information on the behavioral data see Supplementary Materials.

**TABLE 1:** Behavioral results; In the All Objects part of the exploration phase, infants had all objects at their disposal

Exploration Part	Measure	Normal	High	Variable
All Objects. *Total N = 23*	Object First Touched (novel object‐goal association)	*N* = 18	*N* = 19	*N* = 14
	Successful Target Performance	*N* = 2	*N* = 7	*N* = 6
Target Objects Only. *Total N = 22*	Successful Target Performance	*N* = 9	*N* = 12	*N* = 12

In the Target Objects Only part, infants had access only to the target objects corresponding to the respective goal base.

### 2.6 Analysis of relation between EEG and behavioral data

To investigate whether there is a link between infants’ attentional processing of variable movements in IDAs and their subsequent exploration and learning, we calculated partial correlations. The neural measure (frontal theta power) was correlated separately with each of the behavioral measures, that is with exploration (Object First Touched) in the All Objects Part, and with learning (Successful Target Performance) in the All Objects and Target Objects Only part, while controlling for the other behavioral measures, respectively. All behavioral measures were taken during the time infants could explore the objects together with the goal base matching the variable condition. Due to both the focus of this study being on the variable condition and the limited data spread of frontal theta power at Fz in the high and normal conditions, correlations for these conditions were not included in the main manuscript. However, for completeness, they are available in the Supplementary Materials.

## 3 RESULTS

### 3.1 EEG results

**FIGURE 4:**
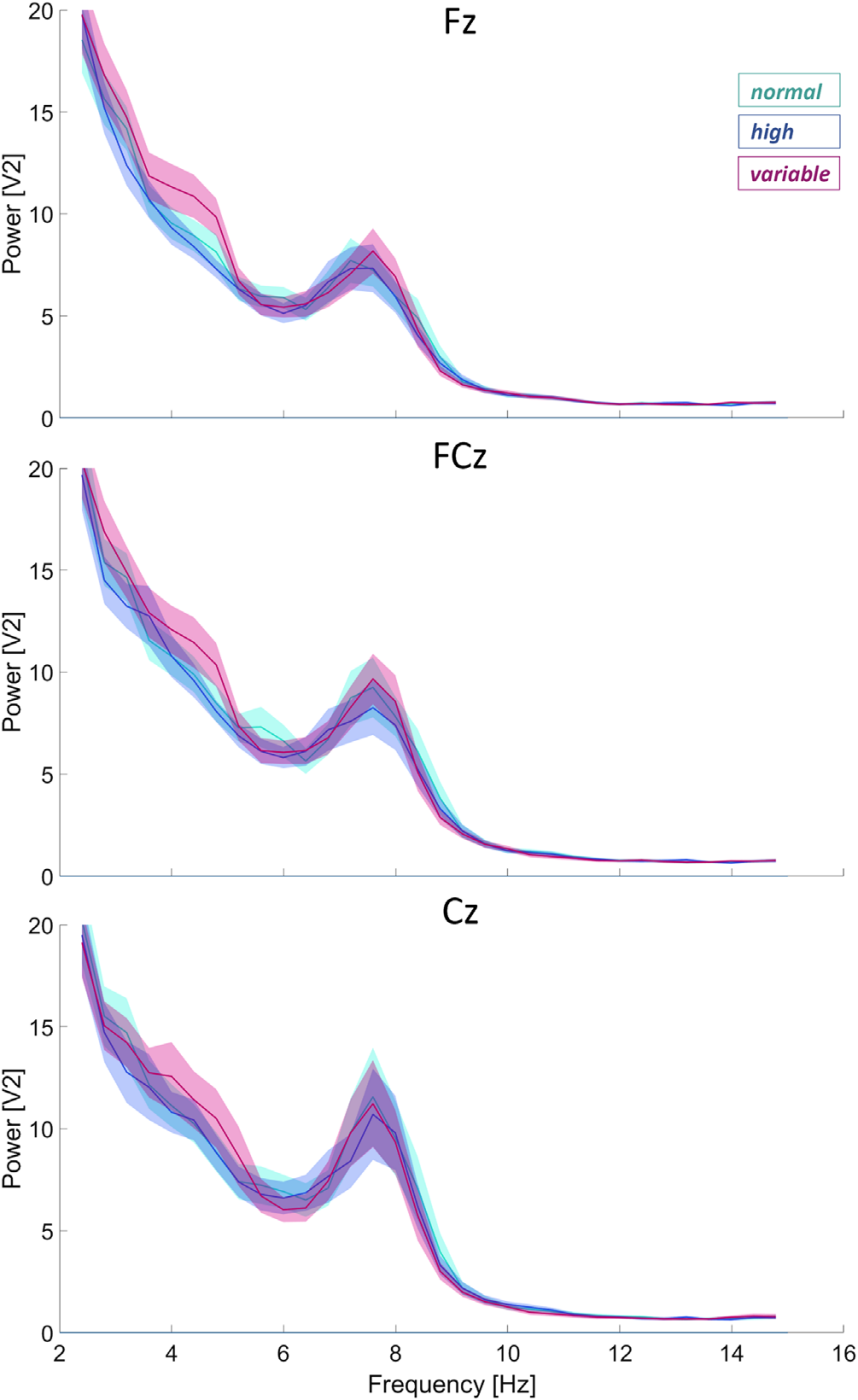
Power as a function of frequency is plotted per condition (normal, high, variable) for electrodes Fz, FCz, and Cz. Mean power values (solid lines) ± 1 SE are displayed in shaded areas for all three electrodes

**FIGURE 5:**
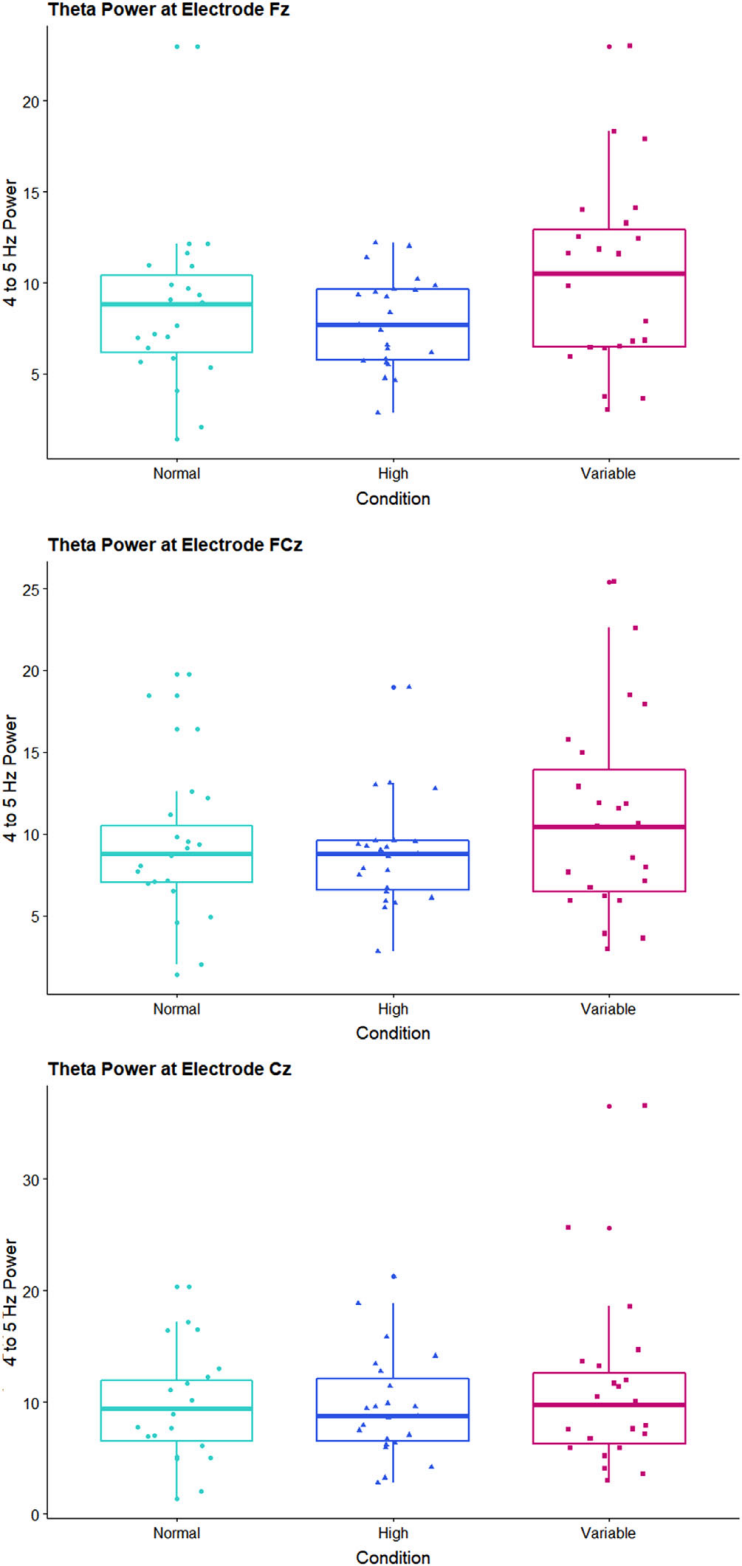
Results represented in boxplots overlayed with individuals’ values. theta power values (4–5 Hz) are displayed as a function of condition (normal, high, variable) for electrodes Fz (top), FCz (middle) and Cz (bottom)

Figure 4 illustrates the grand average power across participants at pre‐defined channels Fz, FCz, and Cz as a function of frequency (3‐30 Hz) and condition (normal, high, variable). In addition to the inherent 1/f distribution of power, the figure shows a frequency‐specific modulation of condition in the theta band (4‐5 Hz). That is, power in the theta band is higher for the variable compared to both the normal and high amplitude movement conditions. This difference appears to decrease from frontal to central sites. Figure 5 shows boxplots of the extracted theta power values per condition for each of the channels. A repeated‐measures ANOVA comparing theta power (4–5 Hz) between conditions yielded a significant difference at channel Fz (*F*(2,44) = 5.00, *p* = .011, partial eta*^2^* = 0.18). At this frontal site, theta power was highest for the variable condition (*M* = 10.35, *SD* = 5.08) and differed significantly from the normal (*M* = 8.53, *SD* = 4.31; *t*(22) = ‐2.318, *p* = .03) and high amplitude conditions (*M* = 7.84, *SD* = 2.54; *t*(22) = ‐2.687, *p* = .01). No evidence for a difference in theta power was found between the normal and high conditions (*t*(22) = 0.951, *p* = .35). At the fronto‐central site FCz, theta power was also descriptively higher for the variable condition (*M* = 10.90, *SD* = 6.00) than the normal (*M* = 9.17, *SD* = 4.52) and high conditions (*M* = 8.82, *SD* = 3.32) but this difference did not reach significance (*F*(2,44) = 2.45, *p* = .09, partial eta*^2^* = 0.10). At central site Cz, no significant difference was detected *(F*(2,44) = 1.15, *p* = .32, partial eta*^2^* = 0.05) between the variable (*M* = 10.96, *SD* = 7.61), normal (*M* = 9.56, *SD* = 4.84) and high (*M* = 6.62, *SD* = 4.69) conditions. Note that we used a Bonferroni‐corrected alpha level of 0.016 to correct for multiple comparisons.

**FIGURE 6:**
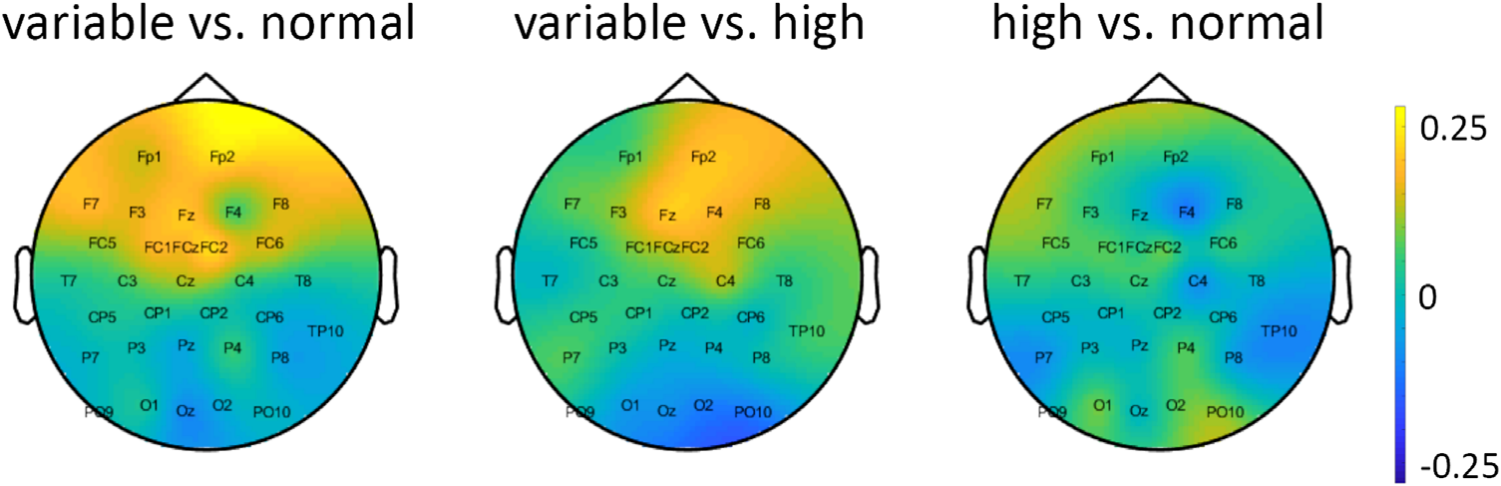
Topographic maps showing the difference in power of the theta frequency range (4–5 Hz) between variable versus normal (left), variable versus high (middle), and high versus normal (right). Warm colors represent more power in theta for the variable (left and middle) and high (right) conditions, respectively

Figure 6 shows the topographic distribution of the theta power difference across the scalp between the variable and normal as well as the variable and high conditions. In line with indications from the three pre‐defined channels, the plot suggests a frontal distribution of the effect. Together, the results provide evidence for an effect in the theta frequency range between conditions such that IDAs with variable movements elicit more frontal theta power than IDAs with normal or high amplitude movements.

### 3.2 Results on the relation between EEG and behavioral data

We examined the relation between neural measures of attention during the variable condition (theta power) and behavioral measures using partial correlations. The results show a relation between higher theta power and successfully performing the target actions in the Target Objects Only part at least once, *r*(18) = ‐0.50, *p* = .025. Moreover, we found that higher theta power was related with touching those objects first that were novel in the context of the given goal in the All Objects part, *r*(18) = ‐0.49, *p* = .028. There was no evidence for a significant relation between theta power and successful target performance in the All Objects part, *r*(18) = −0.36, *p = .12*.

## 4 INTERIM DISCUSSION I

We found that frontal theta was significantly higher, indicating stronger attentional engagement, in the variable compared to both the normal and high amplitude conditions at electrode Fz. This shows that variability in movement amplitude rather than large movements alone engages infants’ attention during infant‐directed actions. Whereas movements with high amplitude are considered prototypical of IDAs (e.g., Koterba & Iverson, 2009; Williamson & Brand, 2014), this evidence supports the idea that it is the variability of movements that parents exploit to draw their infants’ attention to an action. This is consistent with previous findings in IDAs showing that mothers increased the variability in their movements if their infant was unsuccessful in performing the demonstrated action (Fukuyama et al., 2015).

We further examined whether theta power in the variable condition was related to infants’ learning (indexed by correctly performing the target action) and exploration (indexed by novel object‐goal associations). The results show that infants with higher theta power were more likely to successfully perform the target actions and to explore objects that were novel in the context of the given goal. One might have expected that infants with higher theta power first touch the objects corresponding to the goal base rather than objects novel in the context of that goal. Still, the findings fit well with the learning progress framework (Oudeyer et al., 2016), which posits that infants engage in an activity as long as they can still learn from it, but switch to new activities when they cannot learn from the activity anymore (see also Poli et al., 2020). For infants showing higher theta power the input might have fulfilled their learning, which in turn led infants to invest their cognitive resources on exploring novel object‐goal associations. Importantly, all toys (balls, cups, rings) were presented equally often and whether object‐goal associations were shown in variable, high or normal movement demonstrations was counterbalanced across participants. Therefore, this effect cannot be attributed to differences in object or goal saliency per se. In sum, the current exploration effect linking frontal theta power and infants’ first object exploration might reflect habituation processes during the demonstration phase.

### 4.1 What is so special about variability?

Together, the current findings show that movement variability draws infants’ attention and emphasize the role of variability for infants’ attention and learning. That, however, raises the question, what is so special about variability in movement amplitude? To better understand *how* IDAs capture infants’ attention, we examined which information entailed in variability is associated with the observed neural effect.

The variable movement condition differs from the normal and high amplitude movement conditions in multiple ways. First, variable demonstrations are more complex, containing less redundant information than IDAs with constant amplitude movement demonstrations. In a looking time study, Addyman and Mareschal (2013) investigated whether the complexity of visually presented stimulus sequences predicted infants’ looking behavior. Indeed, they found that infants looked longer at sequences that were more complex as indicated by reduced local redundancy. Analogously, this might suggest that it is the stimulus complexity of the variable action demonstrations which is capturing infants’ attention.

Besides being more complex, variable demonstrations are also less predictable, thus more surprising than the constant amplitude demonstrations (normal and high). The role of surprise in dyadic infant‐parent interactions has also been highlighted by research on infant‐directed speech. Note that we refer to surprise in this context as a cognitive construct rather than an emotional state or expression. A computational modelling analysis of variability in infant‐directed speech has shown that when talking to their infants, caregivers use more surprising, less predictable intonation in their speech than when talking to another adult (Räsänen et al., 2018). Given these previous findings, one might expect surprise in the variable IDAs to elicit enhanced attention in infants as indicated by frontal theta increase.

From an information‐theoretic perspective, both the complexity as well as the surprise level are higher in the variable condition than in the other two conditions. Therefore, whether infants are attracted by the complexity or surprise of these stimuli is an open question. Although complexity and surprise both affect the predictability of the stimulus, they make different assumptions on how infants process IDAs. To disentangle these two alternatives, we used a computational modelling approach in a post‐hoc analysis to examine whether complexity or surprise in the current stimuli were predictive of infants’ attentional processing as reflected in frontal theta power.

## 5 COMPUTATIONAL MODELLING INVESTIGATING THE ROLE OF COMPLEXITY AND SURPRISE FOR INFANTS’ ATTENTION TO IDAS

**FIGURE 7:**
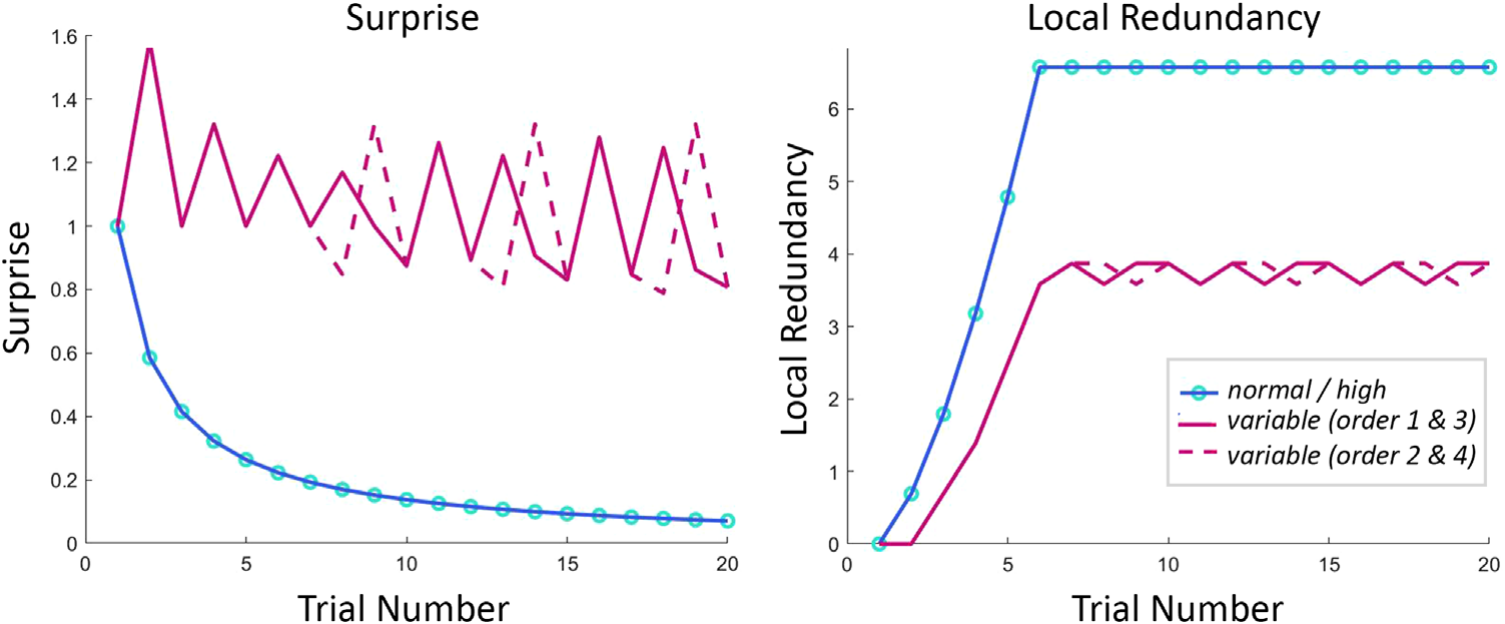
Surprise (left) and Local Redundancy (right) as a function of trial number in example sequences of the normal, high, and variable condition. The variable condition had several counterbalancing orders. The values were computed considering the conditions as independent of each other. Trials of each condition were treated as consecutive events in a sequence of goal‐directed movements making the assumption that infants dissociate the three different actions despite their presentation in separate blocks of videos

We computed the amount of complexity and surprise of each stimulus infants saw, excluding all trials on which infants looked away. As a proxy for complexity, we adopted the measure of local redundancy (Jamieson & Mewhort, 2005) as modified and used by Addyman and Mareschal (2013):

(1) $$R\left(N\right)=\log\left(k_{normal}!\right)\;+\log\left(k_{high}!\right)$$

where *k_normal_* and *k_high_* indicate how many times normal amplitude and high amplitude trials have occurred, and N indicates the maximum number of trials to be considered at each timepoint (i.e., the moving‐window size). Following Addyman and Mareschal (2013), we set *N* = 6. An example of how local redundancy scores change over trials is illustrated in Figure 7. High values of Local Redundancy reflect low complexity in the stimulus. Local Redundancy is higher for normal and high amplitude conditions compared to the variable amplitude condition, thus capturing the difference in complexity between conditions.

As second measure of interest, we computed conditional surprise at each trial as the amount of Shannon Information, I:

(2) $$I\;=log_{2}\;\;p\left(x|X,\alpha \right)$$

where x is the type of movement that occurred in the current trial (high or low amplitude), X consists of all the past actions that have been observed, and α captures the prior expectations at the start of the task. More specifically, the prior expectation of the model at the start is that either a high or a low movement amplitude can occur with equal probability. This can be described in the prior Dirichlet distribution *P*(*p*|α) where all elements of alpha are one, that is, α  =  [1, 1]. This alpha is thus unbiased and expectations can be quickly updated with incoming observations. The past history of all actions X is defined by the number of observations for each event. In other words, how many high and how many low movement amplitudes were observed until the current trial. The likelihood of a high or low amplitude movement to occur at a certain trial is defined by the total number of times this type of movement has occurred in the past in addition to the value of α for that movement type (i.e., 1), divided by the total number of observations up until this point, plus two (i.e., the sum of values of α). For example, this could be used to determine the likelihood that infants will see a high movement amplitude at trial 5 when previously they have seen high, low, low, high, movements (i.e., trial 1 = high, trial 2 = low, trial 3 = low, trial 4 = high, current trial 5 = high). Then, the likelihood of trial 5 being a high movement amplitude is given by the total number of times this type of movement has occurred in the past (i.e., 2), plus alpha for this movement type (i.e., 1), divided by the total number of observations up until this point (i.e., 4 trials), plus two (sum of values of α). In this example, it would thus be: (2+1)/(4+2) = 3/6 = ½. Given the probability of ½ in this example, Shannon information would hence be − *log*_2_ *p*(*x*|*X*,α), so $-log_{2}\;\frac{1}{2}=\;$1. Figure 7 illustrates how Surprise values change across trials for the normal, high, and variable amplitude conditions. While Surprise scores remain rather high and fluctuate on this elevated level in the variable condition, Surprise is approaching zero as trial number increases in the conditions with only normal or high amplitude movements. This indicates that variable IDAs remain surprising while repetitive normal and high amplitude IDAs quickly become predictable to the observer.

Our modeling work allows us to disentangle which of these factors is related to the variation in infants’ theta power. Moreover, it is important to stress that redundancy and surprise measures make very different assumptions on how infants process IDAs. Redundancy is based on stimulus frequency, and thus it assumes that infants keep track of how often a stimulus is presented. Instead, surprise is based on probabilities, which implies that infants make probabilistic models of the world and are surprised when such models are violated.

To test whether we replicate previous research showing condition‐independent increase of theta power over time (Braithwaite et al., 2020), in our analysis we also used the trial number as proxy for time. Importantly, Trial Number did not vary as a function of condition. Then, trial‐based theta power at channel Fz was extracted per participant for each trial and standardized using z‐transformation. Using a Cullen and Frey (1999) to examine our data distribution showed that a lognormal distribution fit the normalized theta power values best. Accordingly, we used generalized linear models (GLMs) to fit our data. The GLMs were fitted using the *gamlss* package (Stasinopoulos & Rigby, 2007) in R. We ran models using z‐transformed values of Local Redundancy, Surprise and Trial Number to estimate their relation to normalized frontal theta power.

The results are represented in Table 2. The model which included Surprise and Trial Number fit the data best (AIC = 2380) and showed a positive relation of frontal theta power with Surprise and positive relation of frontal theta power with Trial Number. This suggests that the higher the levels of surprise in the stimuli, the higher infants’ theta power. Also, the more movements infants saw, reflected in higher Trial Number, the higher their theta power. Any model including Local Redundancy or only individual predictors performed worse (AIC ≥ 2383).

**TABLE 2:** Model comparison including the following variables I = Surprise, LR = Local Redundancy, T = Trial Number

Model	AIC	ΔAIC
I, T	2380	0
LR, T	2383	3
T	2386	6
I	2391	11
LR	2394	14

Lower AIC values indicate better model fit. ΔAIC is computed as the absolute difference between the best model and any other model.

## 6 INTERIM DISCUSSION II

The findings of our post‐hoc computational modeling analysis replicate previous findings of increasing frontal theta power over time (Braithwaite et al., 2020) and show that surprise more so than complexity induced by the variability in the action demonstrations is linked to infants’ higher theta power. The role of surprise in IDAs and infants’ attentional processing is consistent with Event Segmentation Theory (Kurby & Zacks, 2008; Zacks et al., 2007), which explains how we extract meaningful units from action streams. This theory states that, as observers, we use event models to constantly predict what happens next. When a stimulus is perceived as surprising, it leads to updating of the model in order to improve future predictability, also a core idea of the predictive‐processing framework for infant learning (Köster et al., 2020). The temporary increase in processing at surprising moments thereby leads to more robust encoding and memory formation. While Event Segmentation Theory is focused on predictability troughs inherently present at action boundaries, parents artificially introduce predictability troughs in IDAs when interacting with their infants. This may suggest a similar underlying principle for both highlighting action goals in IDAs and segmenting events from a continuous action sequence (Baldwin et al., 2001). In other words, variable and thereby less predictable movements increase infants’ attentional processing such that their attention level is high at the moment the goal of the action is demonstrated.

Additionally, the current findings are consistent with evidence showing that task‐unrelated, unexpected stimuli increase children's arousal, which, in turn, leads to an improvement of task performance (Pozuelos et al., 2014). Increased movement variability in IDAs can also be interpreted as unrelated (or at most only marginally related) to the achievement of a goal, such as stacking rings on a peg. This additional layer of information tangential to the actual goal may increase the level of perceptual surprise, thereby increasing infants’ arousal. Heightened attention might in turn lead to more efficient learning. Interestingly, the beneficial effect of task‐unrelated stimuli for task performance disappears in late childhood (Pozuelos et al., 2014) and even reverses in adulthood, as the presence of task‐unrelated stimuli hinders adults’ performance on a given task (Wetzel et al., 2012). Hence, surprising stimuli might aid infants’ learning in a unique way.

### 6.1 General discussion

Infants’ attention is drawn to actions that are demonstrated in an infant‐directed way to them (Brand & Shallcross, 2008). Such IDAs also seem to benefit infants’ action learning (Williamson & Brand, 2014). The overarching question of the current research was how IDAs capture infants’ attention and how they affect infants’ subsequent exploration and action learning. In particular, we investigated the role variability plays in infants’ attention to IDAs in an EEG study with 15‐month‐olds. As a neural index of attentional processing, we examined modulations in infants’ frontal theta oscillations during IDAs and their link to subsequent exploration and learning. Consistent with our hypothesis, we show that variability in movements during IDAs rather than large movements per se attracts infants’ attention. Furthermore, infants’ frontal theta power during observation of the variable IDAs was related to their subsequent exploration and performance success of the demonstrated actions. More specifically, infants with higher frontal theta power were more likely to explore objects which were novel in the context of the current goal. For instance, when they had observed balls being moved into a container in the variable condition, they would first explore cups and rings when presented with the container. Furthermore, infants with higher frontal theta power were more likely to perform the target action correctly at least once. This link between frontal theta power of variable movements during IDAs and infants’ learning is consistent with previous research investigating modulations in frontal theta power (Begus & Bonawitz, 2020). Together, these results provide evidence suggesting an important role of variability in drawing infants’ attention to and thereby foster their learning from IDAs.

To examine what is special about the information conveyed in the variable condition, we quantified the levels of surprise and complexity of each goal‐directed movement in a post‐hoc computational modelling analysis. The results not only replicate previous findings showing that frontal theta power increases with time (Braithwaite et al., 2020) but also demonstrate the key role of variability‐induced surprise in increasing infant attention via theta power modulation.

### 6.2 Neural processes: Open questions and future directions

Based on our modeling findings, we might assume that surprise in movement variability elicits frontal theta power synchronization. This synchronization, in turn, leads to higher power during the subsequent goal attainment. The question arises whether the attentional benefit gained from the induced surprise has a temporal limitation and whether this limitation is based on the rhythm of theta and the size of the surprise. For example, would the time‐window during which theta power is increased last longer if the surprise value was higher? Previous findings with adults suggest that the surprise systematically affects the magnitude of the theta increase (see e.g., Mas‐Herrero & Marco‐Pallarés, 2014). Also, one might speculate that the frequency range of the oscillations determines the duration needed to return to a baseline level activity. These constraints in turn will have implications for teaching, both in terms of the degree of variability used and the temporal vicinity between the use of variability and the relevant aspect of the action. Future investigations are needed to address this question by systematically varying surprise magnitude and temporal arrangement between the (goal‐unrelated) variability and the following (goal‐related) action unit.

Besides this, little is known about potential maturational effects of the medial prefrontal cortex (mPFC), which is the proposed neural generator of frontal theta oscillations (Ishii et al., 1999). Indeed, mPFC significantly develops throughout early childhood (Casey et al., 2000). Questions include, for instance, whether structural maturation of this brain area underlies changes in processing of surprise and whether there are beneficial effects of variability for task performance. Interestingly, despite the maturational changes of mPFC in the first years of life, research suggests that this area is already crucially involved in social‐cognitive abilities from early on in infancy (Grossmann, 2013). Thus, it remains up to future research to unravel whether this might suggest any functional changes in processing surprise across development. Interdisciplinary research combining strengths of cognitive neuroscience techniques like fMRI and EEG as well as longitudinal developmental research is needed to address these questions.

### 6.3 Implications for teaching novel actions

Assuming that surprise is guiding infants’ attention and driving their learning during IDAs several implications arise. By introducing variability (and thus surprise) in the movement that precedes the goal of the action, parents can utilize the attentional and mnemonic effects of surprise to teach their infant new actions more effectively. This might also explain the prominent use of repetitions in IDAs. Repetitions are omnipresent in parental IDAs (Brand et al., 2002; van Schaik et al., 2020). They may serve two purposes at once, (1) showing the consistency of the goal of the action while, and (2) allowing for variability across repetitions in the goal‐unrelated movement of the IDAs.

Another important consideration regards individual differences in surprise levels, since the level of surprise is based on the prior probability distribution. In other words, what is surprising to an infant may largely depend on the infant's prior experience. For instance, when an infant observes the same actions frequently, like lifting a cup in the same way over and over again, any deviation from that action will elicit surprise. In contrast, an infant who has hardly ever seen a particular movement or has had experience with variable movements, will react to the same action with a different level of surprise. When trying to optimize teaching of a new action to an infant it might therefore be crucial to individually adapt the degree of variability to induce surprise and thus increase attention appropriately. Parents, who typically know a large extent of their infants’ prior experiences, have a good prerequisite to estimate their infant's surprise level to a particular situation. For people less familiar with an infant the information on infants’ prior experience is not necessarily available. Still, a first behavioral indication of surprise (like changes in pupil size; Lin et al., 2018; Preuschoff et al., 2011) might help tune and adjust the IDAs to the individual infant. Another indicator allowing to dynamically adjust to the individual infant is their behavioral performance as an index of learning. In fact, Fukuyama et al. (2015) showed that parents spontaneously introduce more variability in their movement profile when their infant's behavioral performance is unrelated to the demonstrated action. This suggests that caregivers implement the use of variability as an attentional tool. While surprise induced by variability in IDAs certainly is not an exclusive means to draw infants’ attention to an action, it seems both effective and practical in natural dyadic interactions with infants.

In which aspect of their behavior caregivers introduce variability is another interesting point to consider. In other words, how specific are the current findings to movement amplitude? For instance, in the current design, as in everyday life, variability in movement amplitude covaries with variability in movement duration. This is not surprising, as a larger movement typically takes longer to perform. Change in movement amplitude has been highlighted as one of the characteristic features of IDAs, but also other kinematic features of IDAs have been identified (see e.g. van Schaik et al., 2020). We may speculate that it is the surprise induced by variability in input from the social partner more generally that is driving infants’ attention, rather than this effect being limited to a specific behavior (e.g., movement amplitude). Yet, this remains an open question for now.

Whereas during parents’ action demonstrations, certain parts of the actions are performed with variations, it is noteworthy that in a different context, dyadic interactions with infants benefit from the opposite, namely, predictability. That is, success in joint action coordination is achieved by making one's actions temporally predictable (Vesper et al., 2011). This research by Vesper et al. (2011) with adults suggests that reducing one's movement variability is used as strategy for successful coordination with another person. Hence, when trying to coordinate with an infant, predictability in action performance is likely a more promising approach than introducing more variability.

## 7 CONCLUSIONS

Together, our findings show that the use of variability in movements induces surprise which in turn serves as attentional tool during IDAs and which may have beneficial downstream effects for infants’ action learning. As such, these findings advance our understanding of *how* IDAs capture infants’ attention and how they affect infants’ subsequent exploration and action learning. Making use of variability to elicit surprise might thus be a promising teaching tool to increase attention and foster memory formation.

## ETHICS STATEMENT

The current study adheres to the Declaration of Helsinki and was approved by the local ethics board.

## CONFLICT OF INTEREST

The authors declare no competing interests.

## AUTHOR CONTRIBUTIONS

Marlene Meyer, Johanna E. van Schaik and Sabine Hunnius jointly developed the study concept and design. Marlene Meyer and Johanna E. van Schaik collected the data. Marlene Meyer and Johanna E. van Schaik performed EEG data analyses, and Marlene Meyer and Francesco Poli performed computational modelling analyses. All authors interpreted the data. Marlene Meyer drafted the manuscript and all authors contributed critical revisions. All authors approved the final version of the manuscript for submission.

## Supporting information

Supporting Information

## Data Availability

The data and stimulus material are organized according to the BIDS standard and shared in the Donders Repository https://doi.org/10.34973/0egj-ky51. Moreover, the EEG data processing scripts are publicly available on GitHub via https://github.com/marlenemeyer/EEG_pipeline_variability_in_infant_directed_actions.
