# Supplementary material for: How infant‐directed actions enhance infants’ attention, learning, and exploration: Evidence from EEG and computational modeling
Source: Dev Sci. 2022 Apr 7;26(1):e13259. doi: 10.1111/desc.13259 (PMC10078262; doi:10.1111/desc.13259)
Supplement: Supplementary file 1 — Supporting Information [file DESC-26-0-s001.docx]

***Supplementary Materials***

**How infant-directed actions enhance infants’ attention, learning, and exploration:**

**Evidence from EEG and computational modeling**

**Marlene Meyer^1,2*^, Johanna E. van Schaik^3^, Francesco Poli^1^ & Sabine Hunnius^1^**

**^1^**Donders Institute for Brain, Cognition and Behaviour, Radboud University Nijmegen, The Netherlands

**^2^**Department of Psychology, University of Chicago, USA

^3^Faculty of Behavioural and Movement Sciences, Vrije Universiteit Amsterdam, The Netherlands

***Supplementary Analyses***

**Behavioral performance**

In the All Objects part of the exploration phase, infants had all objects at their disposal, those associated with a goal base (corresponding target objects) and those associated with other goals (non-corresponding distractor objects). To assess infants’ exploration behavior, we examined which object infants touched first. When presented with the goal base associated with the normal movement condition, 18 out of 23 children touched non-corresponding distractor objects first. For the high movement amplitude condition, 19 out of 23 children touched a non-corresponding distractor object first, and in the variable condition, 14 out of the 23 infants started exploring non-corresponding distractor objects first as indicated by their first touch. Cochran's Q test did not indicate any significant differences between the exploration behavior among conditions*,* χ^2^(2) = 3.00*, p* = .22. It should also be noted that the chance level of touching a non-corresponding distractor object is 2/3.

In addition to the first touch, we investigated how many children spontaneously performed the target action at least once per condition. In the All Objects part, 2 out of 23 infants performed the target action at least once in the normal condition, 7 out of 23 in the high condition and 6 out of 23 in the variable condition. Also for this measure, Cochran's Q test did not show any significant differences between conditions*,* χ^2^(2) = 3.23*, p* = .20. In the Target Objects Only part 12, out of 22 infants (1 missing data point due to fussiness) performed the target action at least once in the variable and high conditions while 9 out of 22 infants did so in the normal amplitude condition. Cochran's Q test did not reveal any significant effects between conditions*,* χ^2^(2) = 1.38*, p* = .50.

**Relation between EEG (normal condition) and behavioral data**

Partial correlations of frontal theta power at channel Fz during the normal amplitude condition and infants’ action learning and exploration behavior for presentation of the goal base associated with this condition. None of the correlations reached significance. More specifically, no significant correlation was evident between theta power and successfully performing the target actions in the Target Objects Only part (*r*(18) = -.16, *p* = .51) or the All Objects part (*r*(18) = .18, *p* = .44). Also there was no evidence for a significant relation between theta power and exploring the objects by touching those first that were novel in the context of the given goal in the All Objects part, *r*(18) = -.36, *p* = .11.

**Relation between EEG (high condition) and behavioral data**

Partial correlations of frontal theta power at channel Fz during the high amplitude condition and infants’ action learning and exploration behavior for presentation of the goal base associated with this condition. None of the correlations reached significance. More specifically, no significant correlation was evident between theta power and successfully performing the target actions in the Target Objects Only part (*r*(18) = -.01, *p* = .97) or the All Objects part (*r*(18) = -.28, *p* = .23). Also there was no evidence for a significant relation between theta power and exploring the objects by touching those first that were novel in the context of the given goal in the All Objects part, *r*(18) = -.35, *p* = .13.
